# Construction and Immunogenicity Evaluation of a Ferritin-Based Nanoparticle Vaccine Displaying the PDCoV Spike Receptor-Binding Domain

**DOI:** 10.3390/vetsci13101094

**Published:** 2026-10-15

**Authors:** Shinuo Cao, Nannan Nie, Li Zhang, Changchun Chen, Mo Zhou, Shanyuan Zhu

**Affiliations:** 1 Jiangsu Key Laboratory for High-Tech Research and Development of Veterinary Biopharmaceuticals, Jiangsu Agri-Animal Husbandry Vocational College, Taizhou 225300, China; 2 College of Veterinary Medicine, Nanjing Agricultural University, Nanjing 210095, China

**Keywords:** porcine deltacoronavirus, ferritin nanoparticle, self-assembling protein, receptor-binding domain, neutralizing antibody

## Abstract

Porcine deltacoronavirus (PDCoV) poses a significant threat to the global swine industry, underscoring the urgent need for effective vaccines. In this study, a self-assembling ferritin-based nanoparticle vaccine displaying the PDCoV spike receptor-binding domain (S-RBD), designated PDCoV-S-RBD-FR, was constructed and evaluated for its immunogenicity and safety. Transmission electron microscopy (TEM) showed that the recombinant PDCoV-S-RBD-FR protein formed spherical nanoparticles with a mean diameter of 25.55 ± 4.39 nm. In mice, immunization with PDCoV-S-RBD-FR elicited antigen-specific IgG responses, with titers reaching 1:40,960 following booster immunization. Significantly elevated serum IFN-γ and IL-4 levels were observed following immunization. Notably, virus neutralization assays demonstrated that the vaccine induced functional neutralizing antibodies (titer 1:128) capable of effectively blocking PDCoV-induced cytopathic effects in vitro. Histopathological examination of the heart, liver, spleen, lungs, and kidneys revealed no significant lesions in immunized mice relative to the PBS control group, suggesting good tolerability in mice. Collectively, these findings indicate that the ferritin-based PDCoV-S-RBD nanoparticle vaccine elicits antigen-specific IgG responses together with elevated serum cytokine levels and functional neutralizing activity in vitro and no detectable histopathological alterations in major organs, supporting its further evaluation as a vaccine candidate against PDCoV.

## Simple Summary

Porcine deltacoronavirus is a highly contagious intestinal pathogen that causes severe diarrhea, vomiting, and dehydration in pigs, with mortality rates reaching 30 to 40 percent in young animals. The absence of an effective commercial vaccine leaves the global swine industry vulnerable to substantial economic losses. This study aimed to develop a novel vaccine candidate using ferritin, a naturally occurring iron-storage protein that self-assembles into hollow nanoscale cages, to display the receptor-binding domain of the viral spike protein on its surface. The recombinant fusion protein was produced in mammalian cells and purified, forming spherical nanoparticles of approximately 26 nanometers in diameter, as assessed by TEM. When administered to mice, the vaccine induced antigen-specific antibody responses with titers reaching 1:40,960 after booster immunization, and generated functional neutralizing antibodies capable of blocking viral infection at a titer of 1:128. The vaccine also triggered elevated levels of immune signaling proteins associated with antiviral defense. Histopathological examination of major organs revealed no significant abnormalities, suggesting good tolerability in mice. These findings suggest that this ferritin-based nanoparticle vaccine is a promising vaccine candidate warranting further evaluation in the natural host for protection against PDCoV, with potential benefits for agricultural sustainability and food security.

## 1. Introduction

Coronaviruses are enveloped, positive-sense single-stranded RNA viruses that have caused significant epidemics in both humans and animals [1,2]. Porcine deltacoronavirus (PDCoV), a highly contagious enteric coronavirus belonging to the genus Deltacoronavirus, was first identified in Hong Kong, China, in 2012, and has since spread globally, with confirmed circulation across multiple swine-producing regions [3,4]. PDCoV is capable of infecting pigs of all ages, although clinical disease is most severe in suckling and weaned piglets [5,6,7]. Infection primarily targets the intestinal epithelium, resulting in villous atrophy and impaired nutrient absorption, and is clinically characterized by acute watery diarrhea, vomiting, dehydration, and weight loss, with reported mortality rates of approximately 30–40% [7,8]. Given its efficient fecal-oral transmission and persistent circulation within swine populations, PDCoV continues to impose substantial economic losses on the global swine industry.

Notably, PDCoV exhibits a broad host range and demonstrates potential for cross-species transmission [9]. In addition to pigs, PDCoV has been shown to infect calves, chickens, and mice under experimental conditions [10,11,12]. More strikingly, a 2021 case report identified PDCoV-specific IgG antibodies in serum samples from three children in Haiti, raising concerns regarding its zoonotic potential and broader public health implications [13]. These findings, together with the ongoing circulation of the virus in swine populations, underscore the increasing threat posed by PDCoV. Until recently, no commercial vaccine was available for PDCoV prevention; however, a monovalent inactivated vaccine based on strain CHN-HN-2014 was approved as a new veterinary drug in China in 2024. Nevertheless, inactivated vaccines induce predominantly systemic humoral immunity with limited mucosal protection, and the ferritin nanoparticle platform evaluated here represents a rapidly adaptable next-generation approach. The continued threat posed by PDCoV, including co-circulation with other enteric viruses and the zoonotic potential noted above, highlights the ongoing need for next-generation vaccine platforms [14,15]. A rational starting point for antigen design is the viral spike (S) glycoprotein, which mediates receptor recognition and is the principal target of the host immune response [16].

The PDCoV spike (S) glycoprotein is a class I fusion protein organized into an N-terminal S1 subunit, responsible for host receptor recognition, and a C-terminal S2 subunit, responsible for membrane fusion [2,17,18]. Within the S1 subunit, the receptor-binding domain (RBD) mediates direct engagement with the host cell receptor; upon receptor binding, conformational rearrangement of the S2 subunit drives membrane fusion and viral entry [19,20]. Owing to its central role in receptor recognition and its status as a principal target of neutralizing antibodies, the S-RBD has been widely recognized as a rational focus for antiviral strategies, including vaccine design [21,22,23]. Optimizing the presentation of S-RBD on an immunogenic scaffold therefore represents a promising strategy for enhancing vaccine efficacy.

Among vaccine platforms, subunit vaccines offer notable advantages, including the absence of infectious risk, minimal adverse reactions, and high antigenic specificity [22,24,25,26]. However, because subunit vaccines typically consist of a single, isolated protein fragment, they often suffer from insufficient immunogenicity, necessitating strategies to enhance antigen presentation and immune activation [27]. Multimerization of antigens on self-assembling nanoparticle platforms has emerged as an effective approach to overcome this limitation. In this class of platforms, ferritin, a self-assembling cage-like protein involved in iron metabolism and widely expressed across diverse organisms, has attracted particular interest [28,29]. Ferritin assembles from 24 subunits into a hollow nanocage with 432 symmetry, exhibiting high thermal and chemical stability, which makes it a robust scaffold for the multivalent display of exogenous antigens [30,31]. Compared with monomeric antigen delivery, a single ferritin nanoparticle can simultaneously present multiple copies of an antigen on its surface, thereby promoting B-cell receptor cross-linking and enhanced antibody responses, as reported in previous studies [32,33]. This platform has been explored for vaccine development against several viral pathogens in humans [34,35] and, more recently, against porcine epidemic diarrhea virus in piglets [28]; however, its application against PDCoV remains limited. Building on this rationale, the present study sought to combine the antigenic specificity of the S-RBD with the multivalent antigen-display capacity of the ferritin nanoparticle platform.

In this study, we designed a ferritin-based nanoparticle vaccine displaying the PDCoV S-RBD antigen, designated PDCoV-S-RBD-FR, and evaluated its expression, self-assembly, and immunogenicity. The recombinant fusion protein was produced in a mammalian expression system and characterized for nanoparticle formation by transmission electron microscopy. The immunogenicity of the vaccine was further assessed in a mouse model, including antigen-specific IgG responses, cytokine production, and neutralizing antibody titers, alongside histopathological evaluation of major organs to confirm safety. This work aims to provide a novel antigen-display strategy for PDCoV vaccine development and to explore the broader potential of ferritin-based nanoparticle platforms in veterinary vaccinology. Furthermore, by demonstrating a genetically fused, mammalian-expressed antigen-ferritin format compatible with oil-in-water emulsion adjuvants, this study defines a distinct and manufacturable route within the growing family of ferritin-based veterinary nanoparticle vaccines.

## 2. Materials and Methods

### 2.1. Ethics Statement

All animal experiments in this study were conducted in accordance with the Laboratory Animal Management Rules of the People’s Republic of China and were approved by the Laboratory Animal Ethics Committee of Jiangsu Agri-animal Husbandry Vocational College (approval no. JSAHVC-2025-108).

### 2.2. Cells and Animals

HEK293T, PK-15, and ExpiCHO cells were maintained in our laboratory. HEK293T and PK-15 cells were cultured in Dulbecco’s Modified Eagle’s Medium (DMEM) supplemented with 10% fetal bovine serum (FBS) and 1% penicillin-streptomycin (100 U/mL penicillin, 100 μg/mL streptomycin) at 37 °C in a humidified atmosphere containing 5% CO_2_. ExpiCHO cells were cultured in CHO Grow^®^ CD medium at 37 °C in a humidified atmosphere containing 8% CO_2_. Female ICR mice (6 weeks old) were purchased from Jiangsu Xietong Pharmaceutical Bioengineering Co., Ltd (Taizhou, China).

### 2.3. Construction and Expression of the PDCoV S-RBD-Ferritin Eukaryotic Expression Plasmid

The S-RBD sequence (residues 302–421 of the spike protein) of PDCoV strain CH/XJYN/2016 (GenBank accession no. AVZ61082.1) was fused to the N-terminus of ferritin via a flexible (G4S)_3_ linker (GGTGGCGGCGGCTCTGGTGGCGGCGGCTCTGGTGGCGGCGGCTCT), with a Kozak sequence, the GT6 signal peptide, and a 10× His tag introduced upstream of the fusion sequence. An *EcoR*I restriction site was appended at the 5′ end, while a stop codon and a *Hind*III restriction site were incorporated at the 3′ end. The designed sequence was commercially synthesized and cloned into the pCAGGS expression vector. The resulting recombinant plasmids, designated pCAGGS-PDCoV-S-RBD-FR and the ferritin-only scaffold control plasmid pCAGGS-FR, were validated by double restriction enzyme digestion and sequencing.

HEK293T cells were seeded in six-well plates 24 h prior to transfection and transfected upon reaching 80–90% confluence. Transfection complexes were prepared by combining 2 μg plasmid DNA with 4 μL X-tremeGENE HP DNA transfection reagent, followed by gentle mixing and incubation at room temperature for 20 min to allow complex formation. The complexes were then added dropwise to the culture medium, and cells were incubated at 37 °C in a humidified atmosphere containing 5% CO_2_. After 6 h, the medium was replaced with fresh DMEM supplemented with 2% FBS and 1% penicillin-streptomycin, and cells were cultured for an additional 48 h before harvesting by centrifugation (1200× *g*, 5 min) to obtain cell pellets.

Cell pellets were resuspended in RIPA lysis buffer and lysed by ultrasonication. Following centrifugation, the soluble supernatant and insoluble fractions were collected separately, with the insoluble fraction further solubilized in lysis buffer containing 8 M urea. Lysates (20 μL) were mixed with 4 μL of 6 × SDS-PAGE loading buffer and denatured at 100 °C for 10 min. Recombinant protein expression was analyzed by reducing SDS-PAGE, followed by transfer onto a polyvinylidene difluoride (PVDF) membrane and Western blot detection using an HRP-conjugated mouse anti-His tag monoclonal antibody. Immunoreactive bands were visualized with an ECL Plus chemiluminescent substrate and imaged using a gel imaging system.

### 2.4. Construction of Lentiviral Packaging Vectors and Production of Recombinant Lentiviruses

The lentiviral shuttle plasmids were constructed based on the fusion sequence described in Section 2.3. Specific primers were designed to amplify the PDCoV-S-RBD-FR and FR fragments, introducing EcoRI and BamHI restriction sites at the 5′ and 3′ ends, respectively, while the Kozak sequence, GT6 signal peptide, flexible linker, and 10× His tag remained unchanged. The amplified fragments were double-digested with EcoRI and BamHI and subsequently cloned into the similarly digested pLVX-IRES-ZsGreen1 vector by ligation. The recombinant plasmids were transformed into competent E. coli cells, and positive clones were screened by colony PCR and further verified by double restriction enzyme digestion and sequencing. The validated recombinant plasmids were designated pLVX-PDCoV-S-RBD-FR and pLVX-FR and used for subsequent lentivirus packaging.

Recombinant lentiviruses were generated using a three-plasmid packaging system, in which the constructed shuttle plasmids (pLVX-PDCoV-S-RBD-FR and pLVX-FR) were mixed with the packaging plasmids psPAX2 and pMD2.G at a mass ratio of 3:2:1, followed by the addition of PolyJet™ transfection reagent at a plasmid DNA-to-reagent ratio of 1:2. After incubation at room temperature for 20 min, the transfection mixture was added to HEK293T cells. At 48 h post-transfection, transfection efficiency was assessed by fluorescence microscopy, and the culture supernatant was harvested, clarified by centrifugation, and filtered through a 0.45 μm membrane. The filtrate was concentrated 10-fold by ultrafiltration (4500× *g*, 15 min, 4 °C), and the concentrated lentivirus was aliquoted for titer determination or stored at −80 °C for subsequent use. Lentiviral genomic RNA was quantified using the Lenti-X qRT-PCR Titration Kit (Takara Bio, Dalian, China; catalog no. 631235), and RNA copy concentrations were converted to infectious titers (TU/mL) using an equivalence of 200 genome copies per TU.

### 2.5. Expression and Purification of Recombinant Fusion Proteins in ExpiCHO Cells

ExpiCHO cells were cultured to the logarithmic growth phase (viability > 95%) and transduced with recombinant lentivirus at a multiplicity of infection (MOI) of 20, with polybrene added at a final concentration of 8 μg/mL to enhance transduction efficiency. The required virus volume for transduction was calculated as (cell number × MOI) ÷ titer (TU/mL). Following gentle mixing, the cells were incubated overnight at 37 °C in a shaking incubator with 8% CO_2_ and agitation at 125× *g*. At 48 h post-transduction, transduction efficiency was assessed by fluorescence microscopy, and the transduced cells were subsequently expanded for large-scale culture. After expansion, the cell suspension was centrifuged (1200× *g*, 5 min, 4 °C), and the supernatant and cell pellets were collected separately, with cell samples prepared as described in Section 2.3.

Recombinant proteins were purified from the urea-solubilized cell pellet fraction under denaturing conditions using HisSep Ni-NTA MagBeads. Briefly, the pellet fraction solubilized in 8 M urea was incubated with pre-equilibrated MagBeads at 4 °C; non-specifically bound proteins were removed by sequential washing with 30 mM imidazole, and the target proteins were eluted with 300 mM imidazole. The eluted proteins were refolded by dialysis against a refolding buffer (50 mM Tris-HCl, 150 mM NaCl, 400 mM L-arginine, and 5% glycerol) with stepwise reduction of urea concentration (6 M, 4 M, 2 M, 1 M, 0.5 M, and 0 M). Insoluble aggregates formed during refolding were removed by centrifugation (12,000× *g*, 30 min, 4 °C), and the soluble fraction was retained. Protein concentrations were determined using a BCA protein assay kit. Owing to partial protein loss during refolding, formal refolding recovery and per-liter yields were not calculated in this study. Two target proteins, PDCoV-S-RBD-FR and FR, were obtained and stored at −80 °C for subsequent use. Expression and antigenic specificity of the purified proteins were verified by SDS-PAGE and Western blotting.

### 2.6. Characterization of Nanoparticle Self-Assembly by Transmission Electron Microscopy

The self-assembly behavior of the purified PDCoV-S-RBD-FR and FR proteins into nanoparticles was subsequently examined by transmission electron microscopy (TEM). Carbon-coated copper grids were subjected to glow discharge treatment (15 mA, 60 s) to render the surface hydrophilic prior to sample loading. A 4 μL aliquot of diluted protein sample was applied to the grid surface and adsorbed for 1 min at room temperature, and excess liquid was gently removed with filter paper to leave a thin, uniform film. The grid was then negatively stained with 4 μL of 3% (*w*/*v*) uranyl acetate solution for 2 min at room temperature, and residual stain was blotted away with filter paper. Following air-drying at room temperature for 15–20 min, the grid was examined by transmission electron microscopy (TEM). Particle diameters were measured on the original micrographs using image-analysis software, and size distributions were compared by unpaired two-tailed *t*-test with Welch’s correction.

### 2.7. Immunization of Mice

Following a 7-day acclimation period, 6-week-old female ICR mice were randomly assigned to three groups (PDCoV-S-RBD-FR, FR, and PBS control; *n* = 5 per group) using a random number table. Outcome assessment was performed in a blinded manner: the investigators performing IFA fluorescence reading and histopathological evaluation were blinded to group allocation. Protein samples were emulsified with MF59 adjuvant at a 1:1 (*v*/*v*) ratio within 2 h prior to immunization, and mice were immunized intraperitoneally on days 0, 14, and 28 with a final dose of 10 μg protein per mouse, corresponding to approximately 190 pmol of PDCoV-S-RBD-FR (~52.5 kDa) and 291 pmol of FR (~34.4 kDa) per dose, in a total volume of 100 μL per mouse; the PBS control group received PBS emulsified with MF59 adjuvant at the same ratio. Longitudinal body weight monitoring was conducted during the 42-day observation period, with mice weighed at 7-day intervals throughout the immunization schedule. Blood samples (100–200 μL) were collected from the tail vein every two weeks (on days 0, 14, 28, and 42), a minimally invasive procedure that was performed without anesthesia in accordance with standard veterinary practice for small-volume sampling in mice. After collection, blood samples were allowed to clot at room temperature for 30 min and then centrifuged at 3000× *g* for 10 min at 4 °C to separate the serum. Serum samples were heat-inactivated at 56 °C for 30 min to destroy complement activity and stored at −80 °C until further analysis. At day 42 post-immunization, mice were euthanized by cervical dislocation. All procedures were conducted in strict accordance with the Laboratory Animal Management Rules of the People’s Republic of China and were approved by the Laboratory Animal Ethics Committee of Jiangsu Agri-animal Husbandry Vocational College (approval no. JSAHVC-2025-108).

### 2.8. Detection of Antigen-Specific IgG Antibody Levels by IFA

Antigen-specific IgG antibody levels in mouse sera at different time points were determined by indirect immunofluorescence assay (IFA). HEK293T cells were seeded in 96-well plates and transfected with pCAGGS-PDCoV-S-RBD or pCAGGS-FR plasmid (0.2 μg per well) upon reaching approximately 80% confluence, with PBS-transfected cells included as a control. At 48 h post-transfection, cells were fixed with 4% paraformaldehyde for 1 h at room temperature, permeabilized with 0.3% Triton X-100, and blocked with blocking buffer for 20 min at room temperature. Sera from mice immunized with PDCoV-S-RBD-FR were tested against HEK293T cells transfected with pCAGGS-PDCoV-S-RBD, whereas sera from mice immunized with FR were tested against cells transfected with pCAGGS-FR. Sera from PBS-immunized mice were tested against both substrates as a negative control.

Sera collected on days 0, 14, 28, and 42 were serially two-fold diluted from 1:20 to 1:40,960 and incubated with the fixed cells for 2 h at room temperature, followed by five washes with PBST. Cells were then incubated with a fluorescein-conjugated goat anti-mouse secondary antibody for 2 h at room temperature, washed with PBST, and mounted with anti-fade mounting medium. Fluorescence signals were visualized under a fluorescence microscope.

### 2.9. Detection of Serum IFN-γ and IL-4 Levels by ELISA

Serum concentrations of interferon-γ (IFN-γ) and interleukin-4 (IL-4) were measured using commercially available ELISA kits according to the manufacturer’s instructions. Briefly, serum samples and serially diluted standards were added to antibody-precoated plates and incubated with HRP-conjugated secondary antibodies. The chromogenic reaction was developed with TMB substrate solution and terminated with sulfuric acid stop solution, and absorbance was measured at 450 nm using a microplate reader. Cytokine concentrations in the samples were calculated from standard curves generated from the corresponding standards.

### 2.10. Determination of Neutralizing Antibody Titers by Virus Neutralization Test

Neutralizing antibody titers in mouse sera were determined by a virus neutralization test (VNT). Serum samples collected on day 42 were serially two-fold diluted from 1:4 to 1:512 and mixed with an equal volume of PDCoV strain CH/XJYN/2016 (200 TCID50/100 μL), a natural recombinant strain belonging to genogroup GII-c (Southeast Asian sublineage), followed by incubation at 37 °C for 1 h to allow serum-virus interaction. The serum-virus mixtures were then inoculated onto PK-15 cells seeded in 48-well plates, and the cells were cultured at 37 °C in a humidified incubator with 5% CO_2_ for 3–5 days. Cell morphology was monitored daily under a microscope, and neutralizing antibody titers were determined based on the presence or absence of cytopathic effects (CPE). Because the serum volume obtained from tail-vein collection was limited, neutralizing antibody titers were determined for the subset of mice with sufficient remaining serum after allocation to IgG and cytokine assays (*n* = 3 for the PDCoV-S-RBD-FR group and *n* = 3–4 for the FR and PBS control groups); no animals were excluded from the study, and all mice completed the immunization schedule.

### 2.11. Histopathological Analysis of Major Organs

At day 42 post-immunization, mice from each group were euthanized, and major organs, including the heart, liver, spleen, lung, and kidney, were collected for histopathological examination by hematoxylin and eosin (H&E) staining.

### 2.12. Statistical Analysis

All data were processed and analyzed using GraphPad Prism 8.0, and quantitative results are presented as the mean ± standard deviation (SD). A value of *p* < 0.05 was considered statistically significant (* *p* < 0.05, ** *p* < 0.01, *** *p* < 0.001, **** *p* < 0.0001; ns, not significant). Antibody titers (IgG and neutralizing) were log_2_-transformed before analysis. Serum IgG titers and cytokine concentrations were analyzed by two-way repeated-measures ANOVA with Šídák’s multiple comparisons test. Neutralizing antibody titers at day 42 were analyzed by one-way ANOVA followed by Tukey’s multiple comparisons test. Body weight data were analyzed by two-way repeated-measures ANOVA with Tukey’s multiple comparisons test. The group size (*n* = 5) was based on prior immunogenicity studies of ferritin-based and nanoparticle subunit vaccines in mice employing similar endpoints (endpoint IgG titration, serum cytokines, and virus neutralization tests), in which group sizes of 4–6 animals were sufficient to detect biologically meaningful differences between groups [28,30]. Accordingly, *n* = 5 was adopted as a balance between statistical power and the ethical principle of reducing animal use (reduction, one of the 3Rs). We acknowledge that a formal a priori power calculation was not performed; for the planned studies in the natural host (pigs), a formal power calculation based on the variance observed in the present study will be conducted. Given the small group sizes (*n* = 5), formal tests of normality (e.g., Shapiro-Wilk) and variance homogeneity (e.g., Levene’s test) have limited power to verify ANOVA assumptions; antibody titers were log_2_-transformed prior to analysis to improve distributional symmetry, and the inferential results should be interpreted with this caveat in mind.

## 3. Results

To evaluate the feasibility of PDCoV-S-RBD-FR as a nanoparticle vaccine candidate, we first confirmed the correct construction and expression of the recombinant plasmids and fusion proteins, followed by characterization of nanoparticle self-assembly and a systematic assessment of humoral immunity, systemic cytokine responses, neutralizing activity, and in vivo safety in immunized mice.

### 3.1. Construction and Expression Verification of the Eukaryotic Expression Plasmids

**Figure 1:**
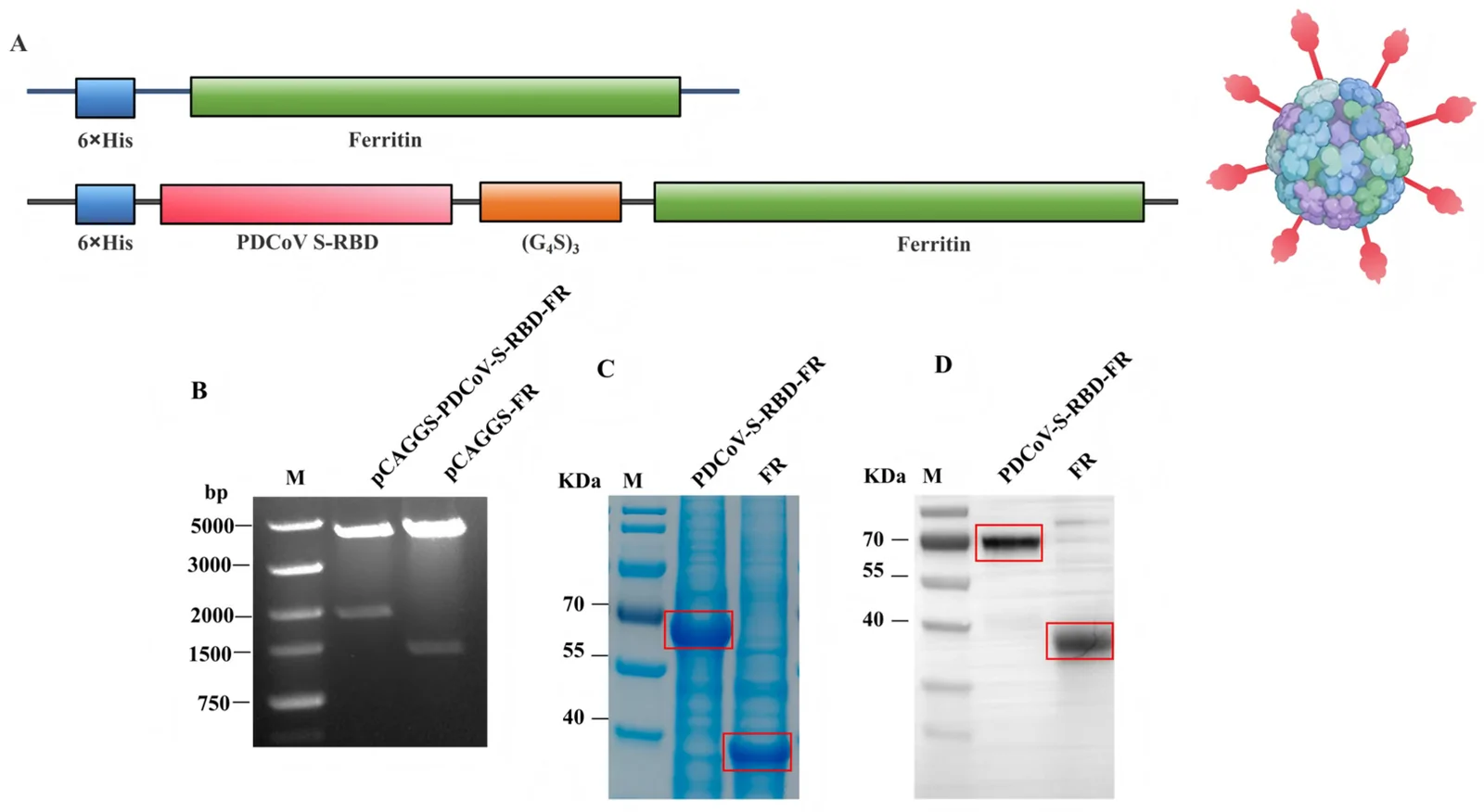
Design, construction, and expression of PDCoV-S-RBD-ferritin recombinant plasmids. (**A**) Schematic representation of the recombinant ferritin scaffold control (FR) and PDCoV-S-RBD-FR fusion protein. The upper diagram shows FR with an N-terminal 10× His tag. The lower diagram depicts the PDCoV-S-RBD-FR construct, in which the PDCoV S-RBD sequence is fused to the N-terminus of ferritin via a flexible (G4S)_3_ linker, with a 10× His tag introduced at the N-terminus. The right panel illustrates the predicted self-assembled nanoparticle structure displaying PDCoV S-RBD spikes on the ferritin surface. (**B**) Restriction enzyme analysis of recombinant pCAGGS expression plasmids. Agarose gel electrophoresis shows pCAGGS-PDCoV-S-RBD-FR and pCAGGS-FR after dual digestion with EcoRI and HindIII, confirming successful insertion of the target fragments. Lane M, DNA molecular weight marker (bp). (**C**) SDS-PAGE analysis of recombinant protein expression in transfected HEK293T cells. Total cell lysates were separated by SDS-PAGE and stained with Coomassie brilliant blue. The red boxes indicate the expected expression bands for PDCoV-S-RBD-FR (predicted 52.5 kDa; apparent ~70 kDa) and FR (predicted ~34.4 kDa; apparent ~40 kDa). Lane M, protein molecular weight marker (kDa). (**D**) Western blot analysis confirming the molecular weights and antigenicity of recombinant proteins. Proteins were probed with anti-His antibody, detecting PDCoV-S-RBD-FR (~70 kDa) and FR (~40 kDa). Lane M, protein molecular weight marker (kDa).

The recombinant eukaryotic expression plasmid pCAGGS-PDCoV-S-RBD-FR and the corresponding ferritin-only scaffold control plasmid pCAGGS-FR were confirmed by sequencing and further verified by double digestion with *EcoRI* and *HindIII* (Figure 1A). Digestion of pCAGGS-PDCoV-S-RBD-FR yielded the expected 4244 bp vector backbone together with a ~1434 bp PDCoV-S-RBD-FR insert, whereas digestion of pCAGGS-FR produced the same vector backbone and a ~1408 bp FR fragment, consistent with the theoretical design (Figure 1B). High-purity plasmids were subsequently prepared at scale and transfected into HEK293T cells. At 48 h post-transfection, total cellular protein was collected and analyzed by SDS-PAGE (Figure 1C)and Western blotting (Figure 1D). Specific bands of approximately 70 kDa and 40 kDa (apparent molecular weights; predicted 52.5 kDa and ~34.4 kDa, respectively) were detected, consistent with expression of PDCoV-S-RBD-FR and FR, confirming successful and efficient expression of the target fusion proteins in mammalian cells. The fusion protein migrated slightly slower than its calculated molecular weight in Coomassie-stained SDS-PAGE, a migration behavior commonly observed for glycoproteins; the identity and approximate size of the full-length protein were therefore confirmed by anti-His Western blotting (Figure 1D).

### 3.2. Generation and Titration of Recombinant Lentiviruses

**Figure 2:**
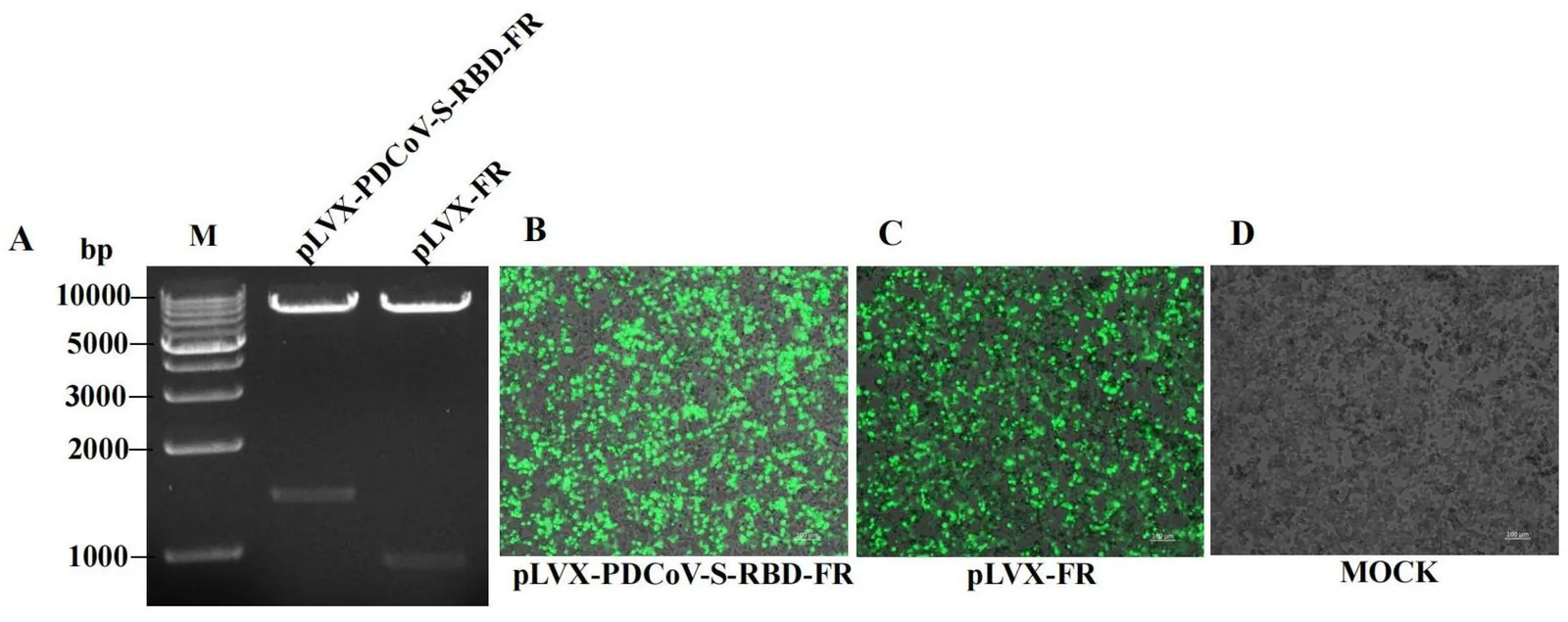
Construction, packaging, and expression validation of recombinant lentiviral vectors. (**A**) Restriction digestion analysis of recombinant lentiviral shuttle plasmids. Digestion of pLVX-PDCoV-S-RBD-FR released the pLVX-IRES-ZsGreen1 backbone (8172 bp) together with the expected ~1434 bp insert, whereas digestion of pLVX-FR released the same backbone and the expected ~950 bp insert, confirming correct construction of both shuttle plasmids. Lane M, DNA marker (bp). (**B**) Fluorescence microscopy of HEK293T cells at 48 h post-transfection with pLVX-PDCoV-S-RBD-FR, showing strong green fluorescence. (**C**) Fluorescence microscopy of HEK293T cells at 48 h post-transfection with pLVX-FR, showing strong green fluorescence. (**D**) Non-transfected MOCK control showing no fluorescence. The presence of green fluorescence in both transfected groups indicates efficient transfection and successful lentiviral packaging.

Having confirmed correct plasmid construction and protein expression in HEK293T cells, we next generated recombinant lentiviruses to enable stable and scalable expression of the target proteins in a suspension cell system. The recombinant lentiviral shuttle vectors were verified by double digestion with *EcoRI* and *BamHI*. Agarose gel electrophoresis showed that digestion of the PDCoV-S-RBD-FR and FR shuttle plasmids released target fragments of approximately 1434 bp and 950 bp, respectively, alongside the common 8172 bp pLVX-IRES-ZsGreen1 vector backbone, consistent with the expected sizes (Figure 2A). The excised fragment (1434 bp) includes the two EcoRI restriction sites and the Kozak sequence; the expressible open reading frame is 1413 bp, encoding a 470-aa fusion protein with a predicted molecular weight of 52.5 kDa. For lentivirus packaging, each shuttle plasmid was co-transfected into HEK293T cells together with the packaging plasmid psPAX2 and the envelope plasmid pMD2.G. At 48 h post-transfection, strong green fluorescence was observed under a fluorescence microscope, confirming high transfection efficiency and successful lentiviral particle production (Figure 2B–D). Viral titers of the harvested PDCoV-S-RBD-FR and FR recombinant lentiviruses were determined using a commercial titration kit, reaching 8.6 × 10^7^ TU/mL and 2.95 × 10^7^ TU/mL, respectively.

### 3.3. Expression and Purification of Ferritin-Based Recombinant Proteins

**Figure 3:**
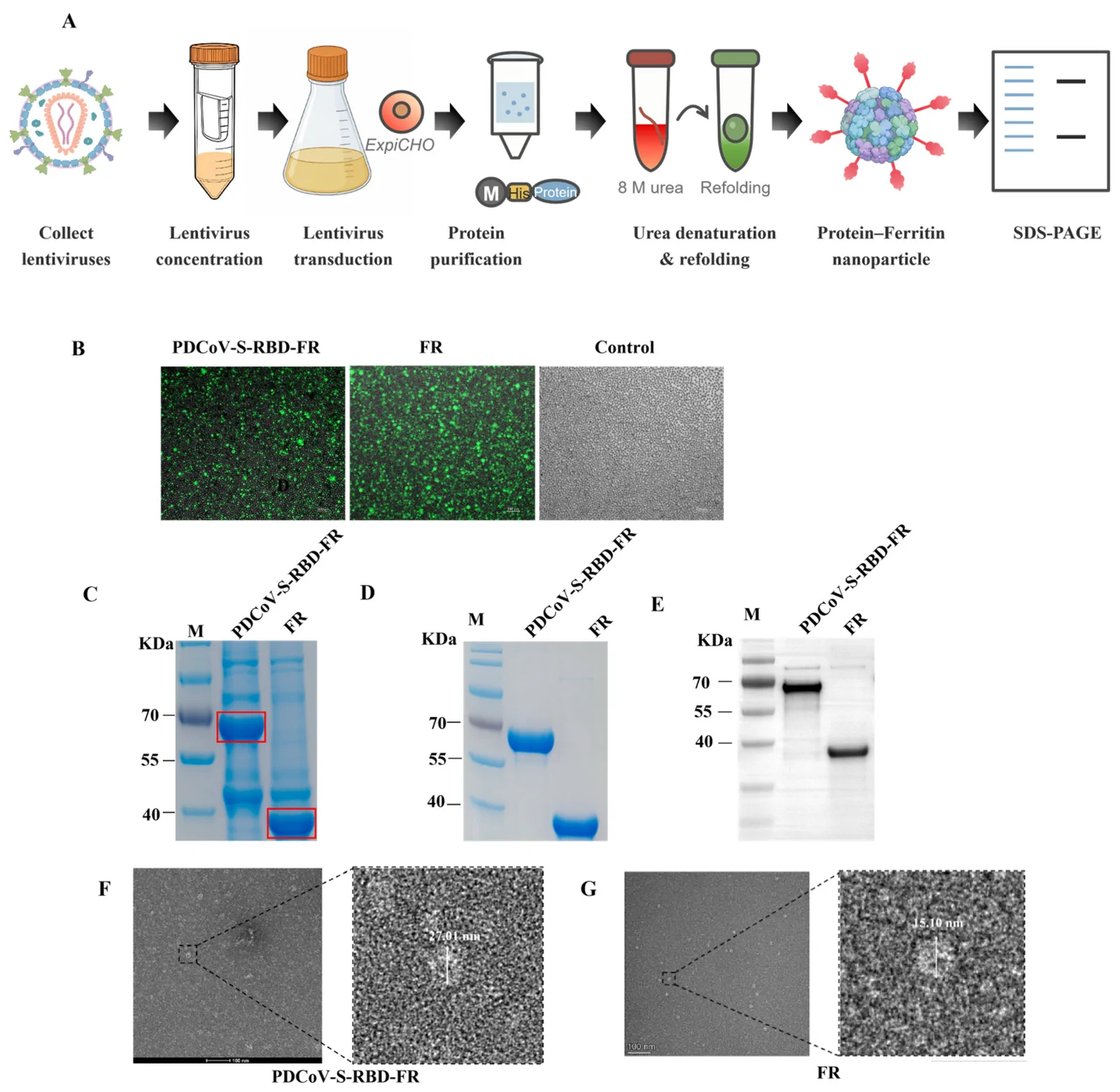
Production and characterization of PDCoV-S-RBD-FR ferritin nanoparticles. (**A**) Schematic workflow for the production of ferritin-based nanoparticles. Recombinant lentiviruses were concentrated and used to transduce ExpiCHO cells for stable protein expression. The target proteins were purified by Ni-NTA affinity chromatography, subjected to urea denaturation and refolding to induce self-assembly into nanoparticles, and finally verified by SDS-PAGE. (**B**) GFP fluorescence detection in ExpiCHO cells at 48 h post-transduction. Strong green fluorescence was observed in PDCoV-S-RBD-FR- and FR-transduced cells, confirming efficient lentiviral gene delivery. No fluorescence was detected in the non-transduced control group. (**C**) SDS-PAGE analysis of crude cell lysates from transduced ExpiCHO cells. Red boxes indicate the target protein bands of PDCoV-S-RBD-FR (predicted 52.5 kDa; apparent ~70 kDa) and FR (predicted ~34.4 kDa; apparent ~40 kDa). Lane M, protein molecular weight marker (kDa). (**D**) SDS-PAGE analysis of purified proteins after His-tag affinity chromatography. Distinct bands corresponding to PDCoV-S-RBD-FR (predicted 52.5 kDa; apparent ~70 kDa) and FR (predicted ~34.4 kDa; apparent ~40 kDa) were observed. Lane M, protein molecular weight marker (kDa). (**E**) Western blot analysis confirming the specificity of purified PDCoV-S-RBD-FR and FR proteins. Lane M, protein molecular weight marker (kDa). (**F**) Negative-stain TEM images of assembled PDCoV-S-RBD-FR nanoparticles; right panel, higher-magnification view. Scale bar, 100 nm. Particle diameters were measured from *n* = 46 particles across multiple fields of view; the size distribution is shown as a histogram (inset), with a mean ± SD of 25.55 ± 4.39 nm (CV = 17%). (**G**) As in (**F**), for FR nanoparticles (*n* = 33; mean ± SD, 16.01 ± 1.78 nm; CV = 11%).

With high-titer recombinant lentiviruses in hand, ExpiCHO suspension cells were transduced to achieve large-scale production and purification of the target fusion proteins (Figure 3A). At 48 h post-transduction, strong green fluorescence was observed under a fluorescence microscope, confirming efficient transduction (Figure 3B). Cell samples were subsequently collected for expression analysis by SDS-PAGE, which revealed specific bands at approximately 70 kDa and 40 kDa (apparent molecular weights; predicted 52.5 kDa and ~34.4 kDa, respectively), respectively (Figure 3C,D); both target proteins were predominantly recovered in the cell pellet fraction. Based on these results, large-scale suspension culture was performed to obtain sufficient cell pellets for protein purification. The expression and antigenic specificity of the purified proteins were subsequently verified by SDS-PAGE and Western blotting with anti-His antibody, respectively (Figure 3E). BCA protein assay determined the final concentrations of purified PDCoV-S-RBD-FR and FR to be 1 mg/mL and 0.5 mg/mL, respectively.

### 3.4. Self-Assembly of Recombinant Proteins into Nanoparticles

Following successful purification, we next assessed whether the purified PDCoV-S-RBD-FR and FR proteins retained the capacity to self-assemble into nanoparticles, a structural feature considered central to their immunogenic potential. The self-assembly of PDCoV-S-RBD-FR and FR proteins into nanoparticles was evaluated by transmission electron microscopy (TEM) and particle size analysis. Particle-size measurements on the original TEM micrographs (*n* = 46 and 33 particles for PDCoV-S-RBD-FR and FR, respectively) revealed mean diameters ± SD of 25.55 ± 4.39 nm (CV = 17%) and 16.01 ± 1.78 nm (CV = 11%), respectively (Figure 3F,G); the size distributions, shown as histograms, differed significantly between the two constructs (unpaired two-tailed *t*-test with Welch’s correction, *p* < 0.0001), and the relatively low CV values indicate a narrow size distribution for both preparations. These results indicate that both recombinant proteins assembled into particulate structures consistent with ferritin nanoparticles.

### 3.5. Antigen-Specific IgG Antibody Responses Induced by Immunization

**Figure 4:**
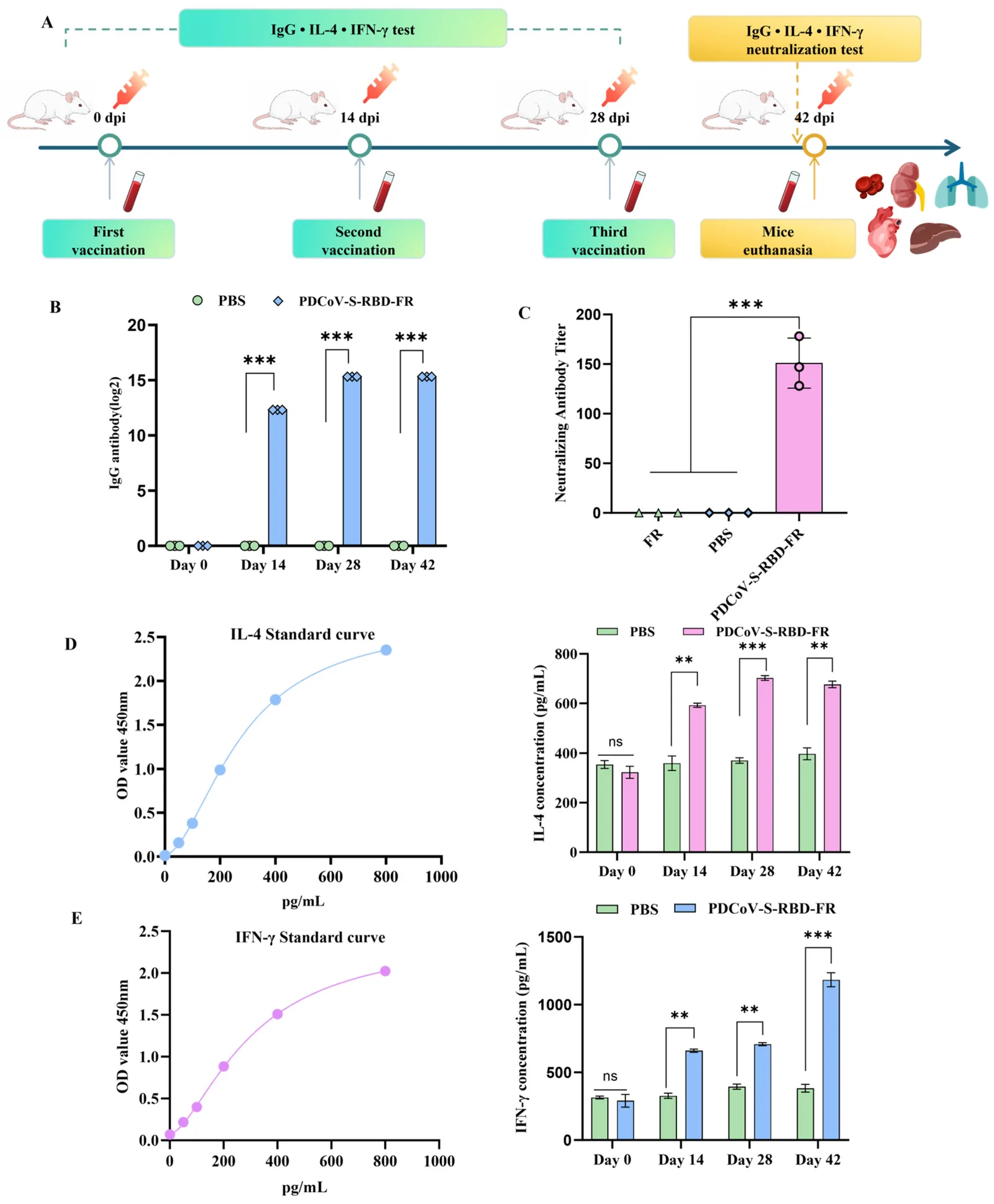
Immunogenicity of PDCoV-S-RBD-FR ferritin nanoparticles in mice. (**A**) Schematic illustration of the immunization and sampling schedule. Six-week-old female ICR mice (*n* = 5 per group) were immunized intraperitoneally on days 0, 14, and 28 with 10 μg of PDCoV-S-RBD-FR or FR protein emulsified 1:1 (*v*/*v*) with MF59 adjuvant; the PBS control group received PBS plus adjuvant. Serum samples were collected on days 0, 14, 28, and 42 post-immunization for IgG, neutralizing antibody, IL-4, and IFN-γ detection. At day 42, mice were euthanized for tissue collection. (**B**) Kinetics of antigen-specific IgG antibody responses. Serum IgG titers (log_2_) were determined by indirect immunofluorescence assay (IFA) on days 0, 14, 28, and 42 post-immunization. Sera from mice immunized with PDCoV-S-RBD-FR were tested against HEK293T cells transfected with pCAGGS-PDCoV-S-RBD, whereas sera from mice immunized with FR were tested against cells transfected with pCAGGS-FR; because the assay substrates differ between groups, the IgG titers of the FR and PDCoV-S-RBD-FR groups are not directly comparable. Sera from PBS-immunized mice were tested against both substrates and showed no specific signal. Data were analyzed by two-way repeated-measures ANOVA with Šídák’s multiple comparisons test. The PDCoV-S-RBD-FR group showed significantly higher IgG titers than the PBS control group (*p* < 0.001). *n* = 5 mice per group; data are presented as mean ± SD. (**C**) PDCoV-specific neutralizing antibody titers at day 42 post-immunization, determined by virus neutralization test (VNT) against PDCoV strain CH/XJYN/2016 (genogroup GII-c, Southeast Asian sublineage). Because of limited serum volume remaining after allocation to IgG and cytokine assays, neutralizing antibody titers were determined for a subset of mice (PDCoV-S-RBD-FR group, *n* = 3; FR group, *n* = 3; PBS control group, *n* = 3); no animals were excluded from the study, and all mice completed the immunization schedule. Data were analyzed by one-way ANOVA followed by Tukey’s multiple comparisons test. The PDCoV-S-RBD-FR group exhibited a neutralizing antibody titer of 1:128, significantly higher than the ferritin-only scaffold (FR) (*p* < 0.001) and PBS control (*p* < 0.001) groups. Data are presented as individual data points with the mean ± SD; data points with identical titers may overlap. (**D**) Serum IL-4 concentrations at different time points post-immunization, measured by ELISA. Left panel, standard curve for IL-4 quantification. Right panel, IL-4 levels were significantly elevated in the PDCoV-S-RBD-FR group compared with the PBS control group at days 14 (** *p* < 0.01), 28 (*** *p* < 0.001), and 42 (*** *p* < 0.001) days post-immunization, with no significant difference at day 0 (ns). (**E**) Serum IFN-γ concentrations at different time points post-immunization, measured by ELISA. Left panel, standard curve for IFN-γ quantification. Right panel, IFN-γ levels were significantly elevated in the PDCoV-S-RBD-FR group compared with the PBS control group at days 14 (** *p* < 0.01), 28 (** *p* < 0.01), and 42 (*** *p* < 0.001) days post-immunization, with no significant difference at day 0 (ns).

Having confirmed nanoparticle formation, we next evaluated whether this structural feature translated into enhanced immunogenicity in vivo, beginning with the antigen-specific humoral response. Antigen-specific IgG responses in mice immunized with PDCoV-S-RBD-FR or FR were assessed by indirect immunofluorescence assay (IFA). Following the immunization schedule shown in Figure 4A, serum samples were collected on days 0, 14, 28, and 42 for antibody detection, with PBS-immunized mice included as the negative control. No antigen-specific IgG antibodies were detected in pre-immune sera (day 0). Following primary immunization, antigen-specific IgG antibodies were clearly detected in both the PDCoV-S-RBD-FR group and the FR scaffold control group as early as day 14, whereas no specific antibody response was observed in the PBS control group throughout the immunization schedule. Following booster immunization, IgG titers increased markedly in the PDCoV-S-RBD-FR group and the FR control group, reaching 1:40,960 in the PDCoV-S-RBD-FR group, and antigen-specific IgG responses were also detected in the FR group against the FR substrate, confirming that the ferritin scaffold is immunogenic. (Figure 4B). The IgG titers for the FR group were measured against a different substrate (FR-transfected cells) than those for the other groups (S-RBD-transfected cells), and that titers across these groups are therefore not directly comparable. Collectively, these results demonstrate that the PDCoV-S-RBD-FR nanoparticle vaccines effectively elicit antigen-specific IgG antibody responses in mice.

### 3.6. Serum IFN-γ and IL-4 Cytokine Responses Induced by Immunization

In addition to humoral immunity, we further assessed systemic cytokine responses elicited by immunization, given its established role in antiviral protection. Serum levels of IFN-γ and IL-4 were measured at different time points post-immunization by ELISA. Except at day 0, both IFN-γ and IL-4 concentrations in the PDCoV-S-RBD-FR group were markedly higher than those in the PBS control group at all subsequent time points (** *p* < 0.01, *** *p* < 0.001, **** *p* < 0.0001) (Figure 4D,E). Of note, the IFN-γ/IL-4 ratio exceeded 1 at day 42 post-immunization; this ratio is reported descriptively only, as no established polarization threshold exists. In addition, because serum cytokines were measured without ex vivo antigen restimulation, these data reflect systemic cytokine levels rather than antigen-specific T-cell responses.

### 3.7. Neutralizing Antibody Responses Induced by Immunization

While IgG and cytokine responses indicate the induction of antigen-specific immunity, functional neutralizing activity is a more direct correlate of protective potential and was therefore evaluated next. A virus neutralization test (VNT) was performed to determine PDCoV-specific neutralizing antibody titers in mouse sera collected at day 42 post-immunization, based on the presence or absence of cytopathic effects (CPE). Cells incubated with serum from the PBS control group exhibited severe CPE, whereas those incubated with serum from the PDCoV-S-RBD-FR group retained normal cell morphology, indicating effective viral neutralization; the corresponding neutralizing antibody titer reached 1:128 (Figure 4C). Collectively, these results demonstrate that, in addition to inducing binding antibodies, the nanoparticle vaccine elicits neutralizing antibody (titer 1:128) that blocked PDCoV-induced cytopathic effects in vitro, suggesting the potential for protective efficacy, which remains to be confirmed in viral challenge studies.

### 3.8. Histopathological Evaluation of Major Organs

**Figure 5:**
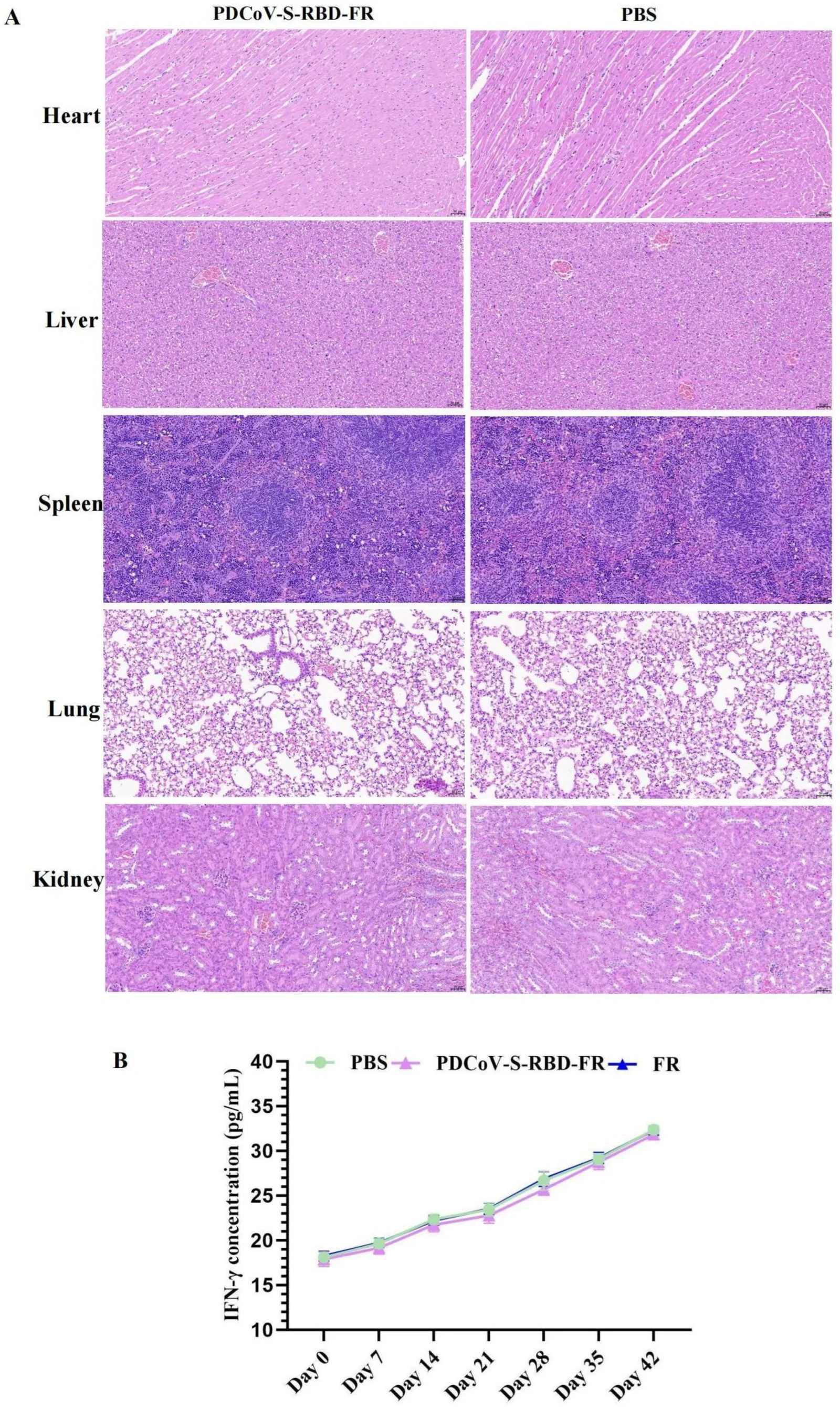
Histopathological analysis and body weight monitoring of mice immunized with PDCoV-S-RBD-FR nanoparticles. (**A**) Representative H&E-stained tissue sections of major organs (heart, liver, spleen, lung, and kidney) from mice at day 42 post-immunization. Tissues from the PDCoV-S-RBD-FR nanoparticle group (left column) and the PBS control group (right column) exhibited normal histological architecture with no significant pathological alterations. (**B**) Longitudinal body weight monitoring of immunized mice during the 42-day observation period. Mice were weighed at 7-day intervals throughout the immunization schedule. Data were analyzed by two-way repeated-measures ANOVA with Tukey’s multiple comparisons test; no statistically significant differences in body weight were detected among the three groups across the 42-day observation period (*p* > 0.05). *n* = 5 mice per group; data are presented as mean ± SD. The absence of significant body weight differences among the PDCoV-S-RBD-FR, FR, and PBS control groups indicates the absence of vaccine-induced systemic toxicity or growth retardation.

Finally, given the favorable immunogenicity profile observed above, the in vivo safety of the vaccine was assessed through histopathological examination of major organs. At day 42 post-immunization, mice were euthanized and major organs were harvested for histopathological evaluation by hematoxylin and eosin (H&E) staining. As shown in Figure 5A, the heart, liver, spleen, lungs, and kidneys of mice in the PDCoV-S-RBD-FR group exhibited no significant pathological alterations compared with those in the PBS control group. These findings indicate that the nanoparticle vaccine, when administered with adjuvant, was well tolerated in this mouse model, with no significant histopathological alterations or body weight changes observed under the tested regimen.

## 4. Discussion

The results presented above demonstrate that the ferritin-based PDCoV-S-RBD-FR nanoparticle vaccine is efficiently expressed, self-assembles into spherical nanostructures, and elicits antigen-specific antibody responses together with elevated serum cytokine levels. These findings warrant further discussion in the context of current PDCoV vaccine development and the broader mechanisms underlying nanoparticle-based immunogenicity enhancement.

Porcine deltacoronavirus (PDCoV) has emerged as a significant enteric pathogen in swine, causing severe gastrointestinal disease and substantial economic losses in the pig industry. Its rapid spread and continuous circulation in pig populations, together with the lack of widely available and effective vaccines, highlight the urgent need for novel immunization strategies. Current vaccine approaches against PDCoV remain limited, particularly in their ability to induce long-lasting protective immune responses. In the present study, a ferritin-based nanoparticle vaccine displaying the PDCoV spike receptor-binding domain (S-RBD) was developed, and its structural characteristics and immunogenicity were systematically evaluated.

Ferritin-based nanoparticles have gained increasing attention as antigen delivery platforms due to their intrinsic ability to self-assemble into highly ordered nanostructures that enable repetitive antigen display. Compared with monomeric antigens, such multivalent nanoparticle platforms are more readily captured by dendritic cells and macrophages, promoting upregulation of costimulatory molecules and thereby enhancing antigen presentation efficiency [36]. This multivalent presentation also mimics the spatial organization of viral epitopes, facilitating B cell receptor cross-linking and germinal center responses [37]. In the present study, the PDCoV-S-RBD-FR fusion protein was efficiently expressed in mammalian cells and assembled into nanoparticles with a mean diameter of 25.55 ± 4.39 nm (*n* = 46) by TEM. Notably, the increased particle size observed upon antigen fusion suggests that incorporation of the S-RBD influences nanoparticle architecture, which may alter antigen accessibility to antigen-presenting cells.

Immunogenicity analysis revealed that the PDCoV-S-RBD-FR nanoparticle vaccine elicited strong and sustained antigen-specific IgG responses. The rapid induction of antibodies following primary immunization and their marked enhancement after booster immunization indicate efficient priming and recall of the humoral immune response. Of note, although the endpoint IgG titers of the PDCoV-S-RBD-FR and FR groups both reached the upper limit of the dilution series (1:40,960) at day 42, the PDCoV-S-RBD-FR groupreached this ceiling earlier during the immunization course, indicating a more rapid and robust humoral response to the S-RBD-bearing nanoparticles. Given that the day-42 titers exceeded the ceiling of the assay, the true endpoint titers at this time point may be higher than reported. Importantly, the antibody response in the PDCoV-S-RBD-FR group was directed against the S-RBD moiety, confirming that the S-RBD antigen is the principal target of the induced antibody response. We note that the FR scaffold control is itself immunogenic, eliciting substantial antigen-specific IgG responses (titers up to 1:40,960 against the FR antigen), confirming that it is an expressed, self-assembling nanoparticle rather than an inert “empty” control. This highlights both the value of the ferritin platform as an immunogenic scaffold and the importance of interpreting vaccine-group responses relative to the FR scaffold control.

Serum cytokines were measured without ex vivo antigen restimulation and therefore reflect systemic cytokine levels rather than antigen-specific T-cell responses; the elevations observed at day 14 may partly reflect innate immune activation by the MF59 adjuvant itself. In addition to humoral immunity, immunization was associated with elevated serum IFN-γ and IL-4 levels, as described above. Importantly, the vaccine also elicited functional neutralizing antibodies, as demonstrated by the virus neutralization test, in which immune sera effectively inhibited cytopathic effects in vitro. It is noteworthy that while antigen-specific IgG titers reached 1:40,960, the neutralizing antibody titer was comparatively modest (1:128), representing an approximately 300-fold gap. This discrepancy is typical of RBD-displaying nanoparticle vaccines. Binding assays such as IFA detect the entire pool of antigen-specific antibodies, including non-neutralizing clones that target immunodominant but non-protective epitopes. In contrast, neutralization assays capture only the subset of antibodies capable of blocking receptor engagement or viral entry. The RBD contains multiple conformational epitopes with varying contributions to neutralization, and the multivalent display on ferritin may enhance B-cell activation to epitopes outside the neutralizing determinants, further widening the binding-to-neutralization ratio. Future work will explore optimization strategies to narrow this gap, such as structure-guided stabilization of the RBD to preferentially present neutralizing epitopes, or the inclusion of additional neutralizing targets such as the S2 subunit. In addition, when formulated with MF59 adjuvant, the vaccine was well tolerated in mice, with no significant histopathological alterations observed in major organs; comprehensive safety evaluation in the natural host will be required. Unlike aluminum-based adjuvants, which are thought to act partly through antigen depot formation, MF59 is an oil-in-water emulsion adjuvant that functions primarily by inducing local innate immune activation at the injection site, promoting the recruitment of monocytes, granulocytes, and dendritic cells, and facilitating monocyte-to-dendritic cell differentiation and antigen transport to draining lymph nodes [38,39]. This mechanism, rather than a depot effect, likely contributed to the robust humoral and cellular immune responses observed in this study, consistent with the established safety and immunogenicity profile of MF59 in licensed human vaccines. It is noteworthy that a recent study reported a ferritin nanoparticle vaccine against PDCoV in which a bacterially expressed RBD dimer was conjugated to ferritin via the SpyTag/SpyCatcher system; that vaccine elicited RBD-specific IgG and neutralizing antibodies and, importantly, provided protection against viral challenge in mice, as evidenced by markedly reduced histopathological lesions [40]. The present study differs from that work in several respects: our S-RBD antigen is genetically fused to ferritin and expressed in a mammalian (ExpiCHO) system, which preserves native post-translational modifications and eliminates the chemical conjugation step; we characterized cytokine kinetics throughout the immunization course rather than at a single time point; and we evaluated the vaccine in combination with the MF59 oil-in-water emulsion adjuvant. At the same time, the protective efficacy demonstrated by Wang et al. underscores the importance of viral challenge experiments; accordingly, challenge studies in the natural host (pigs) are planned in our follow-up work and are acknowledged above as a limitation of the present study.

Despite these encouraging immunogenicity findings, several limitations of the present study warrant consideration before this vaccine candidate can be advanced toward veterinary application. First, a limitation of the present study is the absence of a soluble S-RBD control group administered with the same adjuvant. Although prior studies using identical antigens have consistently shown that ferritin multimerization enhances immunogenicity relative to soluble antigen, a direct within-study comparison will be required to definitively attribute the enhanced responses observed here to nanoparticle display. This comparison is planned in our laboratory. Second, although quantitative particle-size analysis was performed (*n* = 46 and 33 particles), the number of fields examined was limited and orthogonal physicochemical methods such as dynamic light scattering and long-term stability assessment were not applied; more comprehensive characterization will be performed in follow-up studies. Third, a mouse model rather than the natural host (pigs) was used, and immune cell composition, mucosal immune environments, and immune pathways in mice differ from those in swine; mouse models may therefore not fully recapitulate the natural infection process and intestinal pathological changes caused by PDCoV in neonatal piglets. Fourth, viral challenge experiments were not performed in this study, and protective efficacy remains to be validated in vivo. Fifth, because immunization was performed at an equal protein mass (10 μg per mouse) rather than an equal molar dose, the FR group received approximately 1.5-fold more protein moles per dose than the PDCoV-S-RBD-FR group (250 pmol vs. 139 pmol), which may confound the direct comparison between the two groups. In our follow-up studies, dosing will be normalized by molar antigen content. Sixth, neutralizing antibody titers were assessed only at the terminal time point (day 42) and, owing to limited serum volume from serial tail-vein sampling, only for a subset of mice (*n* = 3 for the PDCoV-S-RBD-FR group); the neutralization data should therefore be interpreted as qualitative proof-of-concept evidence, and future studies in the natural host (pigs) will employ larger serum volumes and multiple time points to enable full kinetic profiling. Seventh, cytokine responses were assessed as serum IFN-γ and IL-4 concentrations without ex vivo antigen restimulation; antigen-specific T-cell responses (e.g., ELISPOT or intracellular cytokine staining) were not evaluated and will be included in follow-up studies, together with cytokine measurements for the FR control group. Finally, the expression and refolding conditions used here have not been optimized: partial protein loss occurred during urea-gradient refolding, and refolding recovery and final yields were not quantified; process optimization and comprehensive yield assessment will be performed in future work.

Given that PDCoV frequently co-circulates with other porcine enteric viruses such as PEDV in the field, broader protection against porcine enteric viral diseases will likely require vaccines that address multiple pathogens [41]. The modular, self-assembling scaffold validated in the present study provides a direct rationale for such multivalent designs: the same scaffold that efficiently presented the PDCoV S-RBD could, in principle, accommodate additional pathogen-derived antigens without altering the underlying nanoparticle design. Future work may therefore explore bivalent or multivalent ferritin nanoparticle vaccines co-displaying PDCoV and PEDV antigens on a single scaffold, extending the approach validated here to a broader veterinary application.

## 5. Conclusions

Taken together with the mechanistic considerations discussed above, this study demonstrates that a self-assembling ferritin-based nanoparticle vaccine displaying the PDCoV S-RBD (PDCoV-S-RBD-FR) was successfully constructed, expressed in a mammalian system, and purified by Ni-NTA affinity chromatography with purity verified by SDS-PAGE and Western blotting. TEM analyses showed that the recombinant fusion protein assembled into spherical nanoparticles, consistent with ferritin-based self-assembly for multivalent antigen display. When formulated with MF59 adjuvant and administered to mice, PDCoV-S-RBD-FR elicited antigen-specific IgG responses that increased following booster immunization, with titers reaching 1:40,960 after booster immunization, alongside significantly elevated IFN-γ and IL-4 levels. Notably, the vaccine induced functional neutralizing antibodies capable of blocking PDCoV-induced cytopathic effects in vitro, and no significant histopathological alterations were observed in major organs, with no detectable adverse effects in mice in this study. Collectively, these findings demonstrate that the ferritin-based PDCoV-S-RBD nanoparticle vaccine induces antigen-specific antibody responses together with elevated serum cytokine levels with no detectable adverse effects in mice, providing a novel antigen-display strategy for PDCoV vaccine development and expanding the application of ferritin-based nanoparticle platforms in veterinary vaccinology. Future studies incorporating viral challenge in pigs, the natural host, are warranted to further validate the protective efficacy and translational potential of this vaccine candidate.

## Data Availability

The original contributions presented in this study are included in the article and Supplementary Materials. Further inquiries can be directed to the corresponding authors.

## Supplementary Materials

The following supporting information can be downloaded at: https://www.mdpi.com/article/10.3390/vetsci13101094/s1, File S1 Original Images for Blots.
