# Supplementary material for: Construction and Immunogenicity Evaluation of a Ferritin-Based Nanoparticle Vaccine Displaying the PDCoV Spike Receptor-Binding Domain
Source: Vet Sci. 2026 Oct 15;13(10):1094. doi: 10.3390/vetsci13101094 (PMC13709850; doi:10.3390/vetsci13101094)

Figure S1（B,C,D）

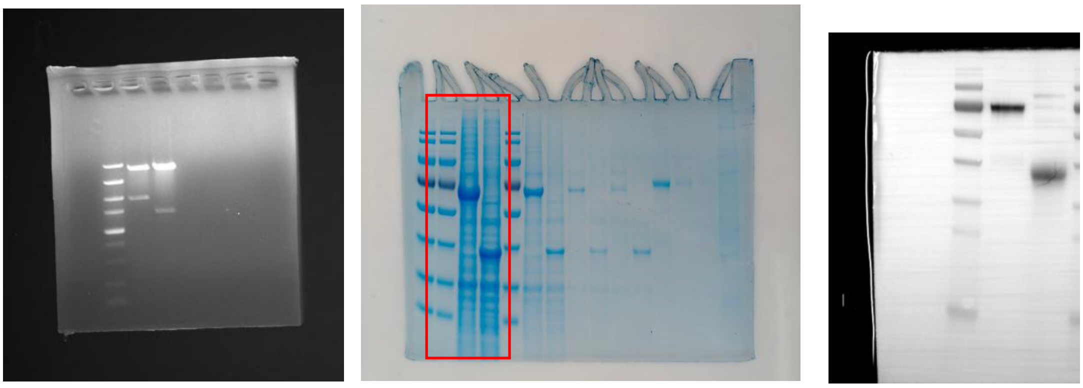

Figure S2A

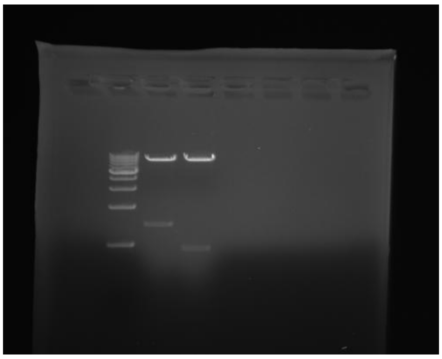

Figure S3（C,D,E）

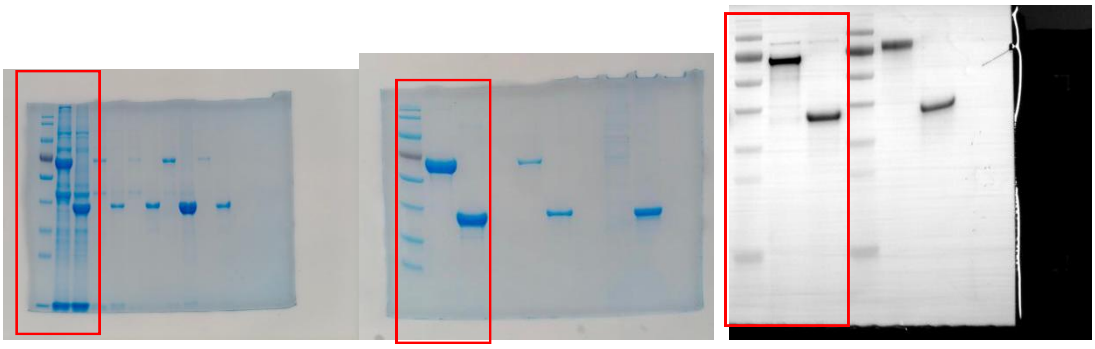

Supplement: Supplementary file 1 [file vetsci-13-01094-s001.zip › vetsci-4583458-supplementary.pdf]
